# Management of a Left Lateral Aortic Paraganglioma During Pregnancy: A Rare Case Report

**DOI:** 10.7759/cureus.19221

**Published:** 2021-11-02

**Authors:** Mohamed Yassine Mabrouk, Rachid Jabi, Laila Bouzayan, Kradi Yassin, Mohammed Bouziane

**Affiliations:** 1 Faculty of Medicine and Pharmacy, Laboratory of Anatomy, Microsurgery and Surgery Experimental and Medical Simulation (LAMCESM) Mohammed 1st University, Department of General Surgery, Mohamed VI University Hospital, Oujda, MAR; 2 Faculty of Medicine and Pharmacy, Laboratory of Anatomy, Microsurgery and Surgery Experimental and Medical Simulation (LAMCESM) Mohammed 1st University, Department of General Surgery, Mohamed VI University Hospital, oujda, MAR

**Keywords:** case report, laparoscopy, retroperitoneal tumor, gravid hypertension, paraganglioma

## Abstract

Paragangliomas are rare neuroendocrine tumors mostly diagnosed in young adults. Their association with pregnancy is even rarer, and their impact is even more serious in the absence of adequate management, which may vitally involve maternal and fetal prognosis. In this report, we present a rare case of a left lateral aortic paraganglioma in a pregnant woman in her third trimester during her 31 weeks of gestation, who consulted for a hypertensive peak; the methoxylated derivatives were positive. An abdominal MRI showed a left lateral aortic mass, suggesting a paraganglioma. After a multidisciplinary discussion, the patient underwent laparoscopic surgical resection of the mass after preoperative medical preparation. Pathological examination confirmed the diagnosis of paraganglioma. The overall stay was six days without any short-term complications, including anything related to fetal viability, with a vaginal delivery at 37 weeks of amenorrhea. The patient was followed up for six months with no complications. We highlight the importance of preoperative medical preparation followed by surgical resection in the framework of a multidisciplinary consultation for an improved maternal-fetal prognosis.

## Introduction

Paragangliomas are rare neuroendocrine tumors that develop from chromaffin cells; in most cases, they involve embryonic neural ridges secreting catecholamines [1]. They are uncommon entities and have an annual incidence rate of 1/100,000 [2]. Their discovery during pregnancy is even rarer and can lead to serious consequences in the absence of adequate treatment [3]. Their clinical manifestations vary depending on the location of the paraganglioma, the size, and the secretion status. Due to their proximity to major vessels and surrounding organs, their diagnosis and treatment may be challenging and require multidisciplinary care and support [4]. In this report, we present a rare case of a left lateral aortic paraganglioma during pregnancy, challenging the diagnosis, the treatment, and altering the prognosis. We have followed the Surgical CAse REport (SCARE) 2020 guidelines in reporting this work [5].

## Case presentation

A 35-year-old female patient, primiparous, and pregnant with an estimated 31 weeks of amenorrhea, consulted our emergency department for a hypertensive peak during pregnancy follow-up associated with palpitations, tinnitus, sweating, and intense headaches. Her medical history was remarkable for high blood pressure for three years under alpha-methyldopa, without any familial history of hypertension. On physical examination, she had an elevated blood pressure of 180/100 mmHg, a normal pulse of 82 beats per minute, a respiratory rate of 26 breaths per minute, and an oxygen saturation of 94% on room air with a capillary blood glucose level of 1.06 g/L. Pulmonary auscultation was unremarkable; the abdominal examination showed a fundal height reaching 28 cm, without any tenderness or palpable mass.

**Figure 1:**
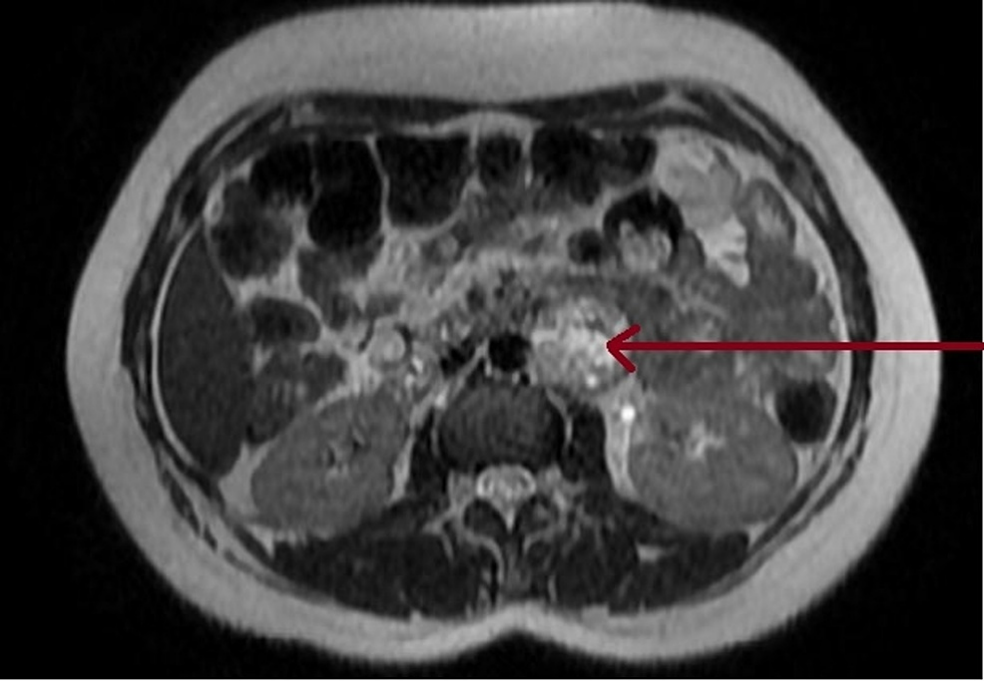
Abdominal MRI The image shows a left-lateralized abdominal juxta-aortic formation, well-limited, with heterogeneous T2 hyperintensity, and measuring 36 x 33 mm (red arrow) MRI: magnetic resonance imaging

As part of the etiological evaluation, routine labs including blood test, liver function, creatinine, and 24-hour proteinuria were within normal ranges; preeclampsia was ruled out. Given the presence of Menard's triad (headaches, sweating, and palpitation) and to obtain the etiological diagnosis, we considered pheochromocytoma or paraganglioma. Urine metanephrines testing was performed, which revealed an elevated normetanephrine level of 4.78 μmol/day (reference value: ≤2.9 µmol/day) and metanephrines level of 5.93 μmol/day (reference value: ≤1.5 µmol/day). Since the patient was pregnant, an abdominal MRI was performed, which showed a left-lateralized abdominal juxta-aortic formation, well-limited in heterogeneous T1 iso-intensity with punctiform areas in spontaneous T1 hyperintensity, and heterogeneous T2 hyperintensity, measuring 36 x 33 mm, suggesting a paraganglioma (Figure 1).

**Figure 2:**
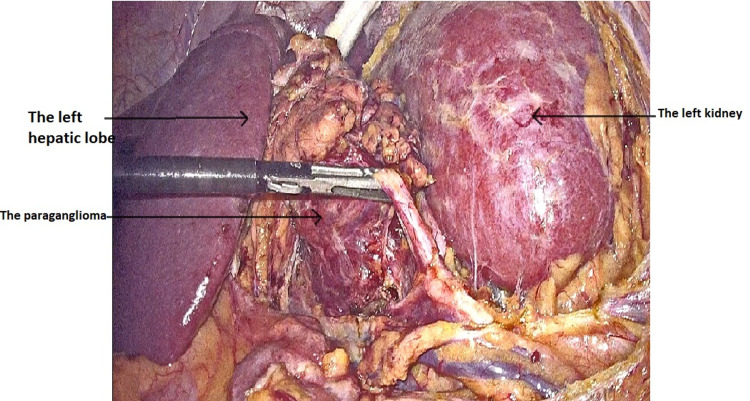
Intraoperative image showing the dissection of the mass from its attachments

**Figure 3:**
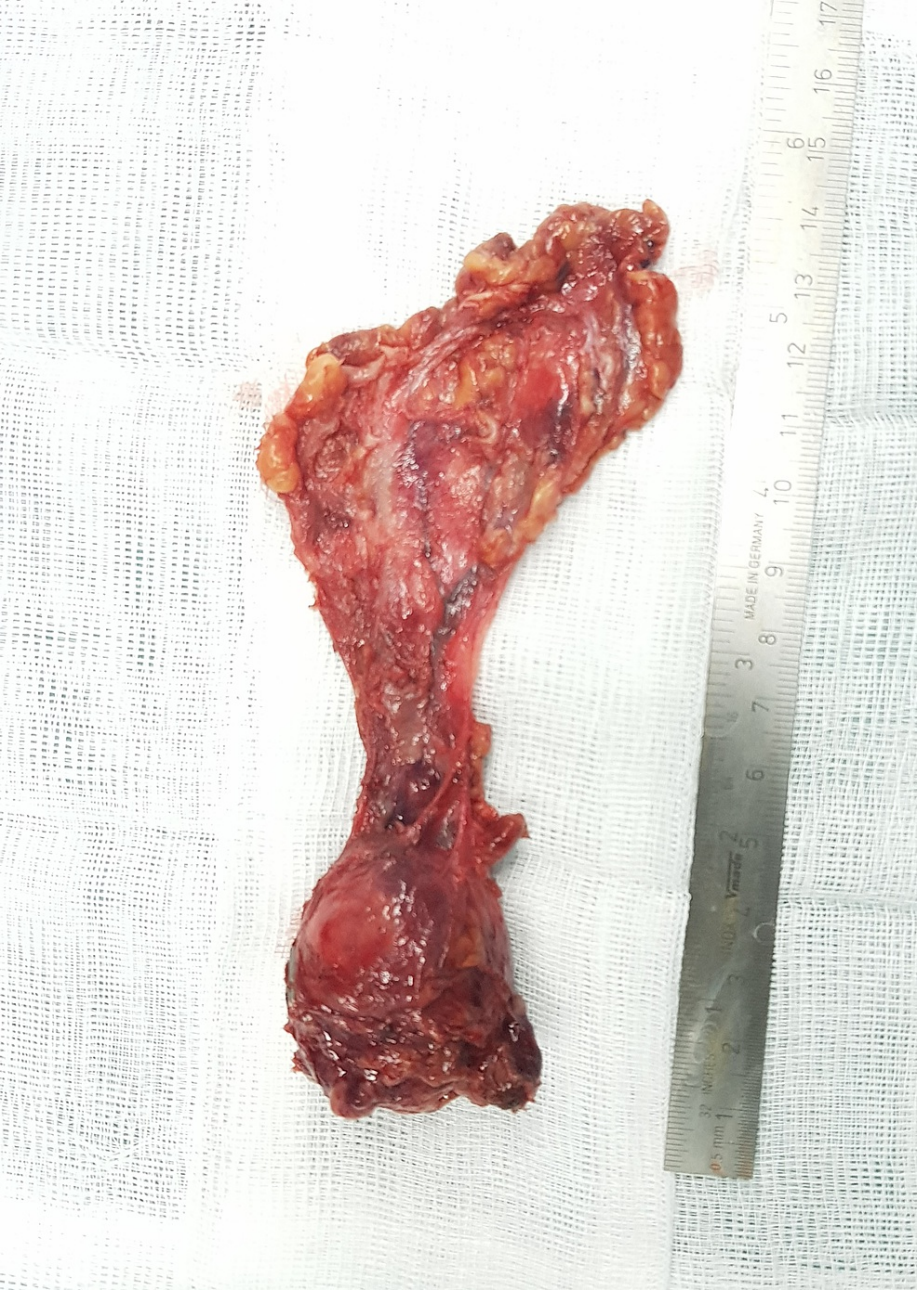
Image showing the resected mass

After a multidisciplinary discussion involving visceral surgeons, anesthesiologists, endocrinologists, and gynecologists, it was concluded that surgery during pregnancy under laparoscopy was the best option given that the patient was symptomatic with a high risk of preeclampsia. After preoperative preparation in the ICU, the patient underwent a laparoscopic surgical resection under general anesthesia (Figures 2, 3).

**Figure 4:**
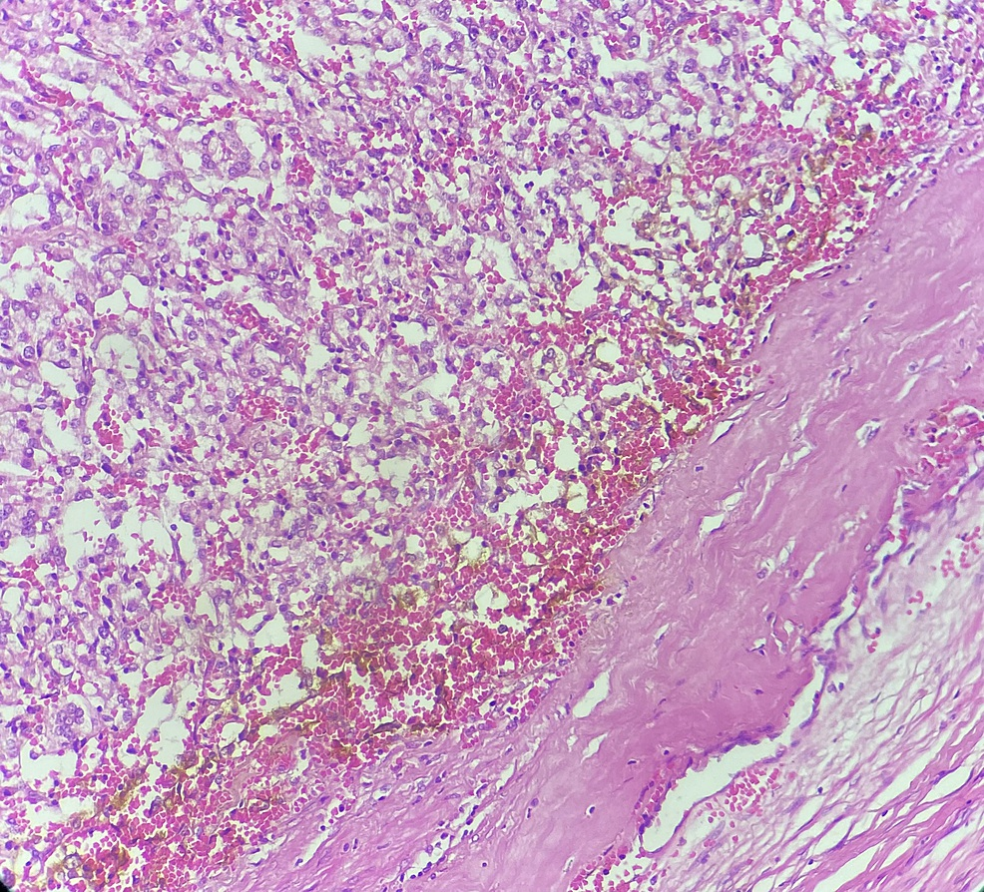
The microphotographic analysis of paraganglioma showing a well-limited tumor proliferation arranged in nests Hematoxylin and eosin (H&E) stain x100

During the manipulation of the tumor, three episodes of arterial hypertension were observed, which were controlled by the reinforcement of the analgesia through reinjections of fentanyl and propofol and the administration of nicardipine. At the end of the operation, the patient underwent a fetoplacental ultrasound, which showed normal fetal cardiac activity. Postoperatively, analgesia, nicardipine, and prophylactic heparin therapy were administered. Pathological examination revealed a well-limited tumor proliferation arranged in nests made of polyhedral cells with a round nucleus and irregular chromatin and granular cytoplasm, These nests were surrounded by cells with an elongated or comma-shaped nucleus with a sparse cytoplasm, the sustentacular cells (Figure 4).

**Figure 5:**
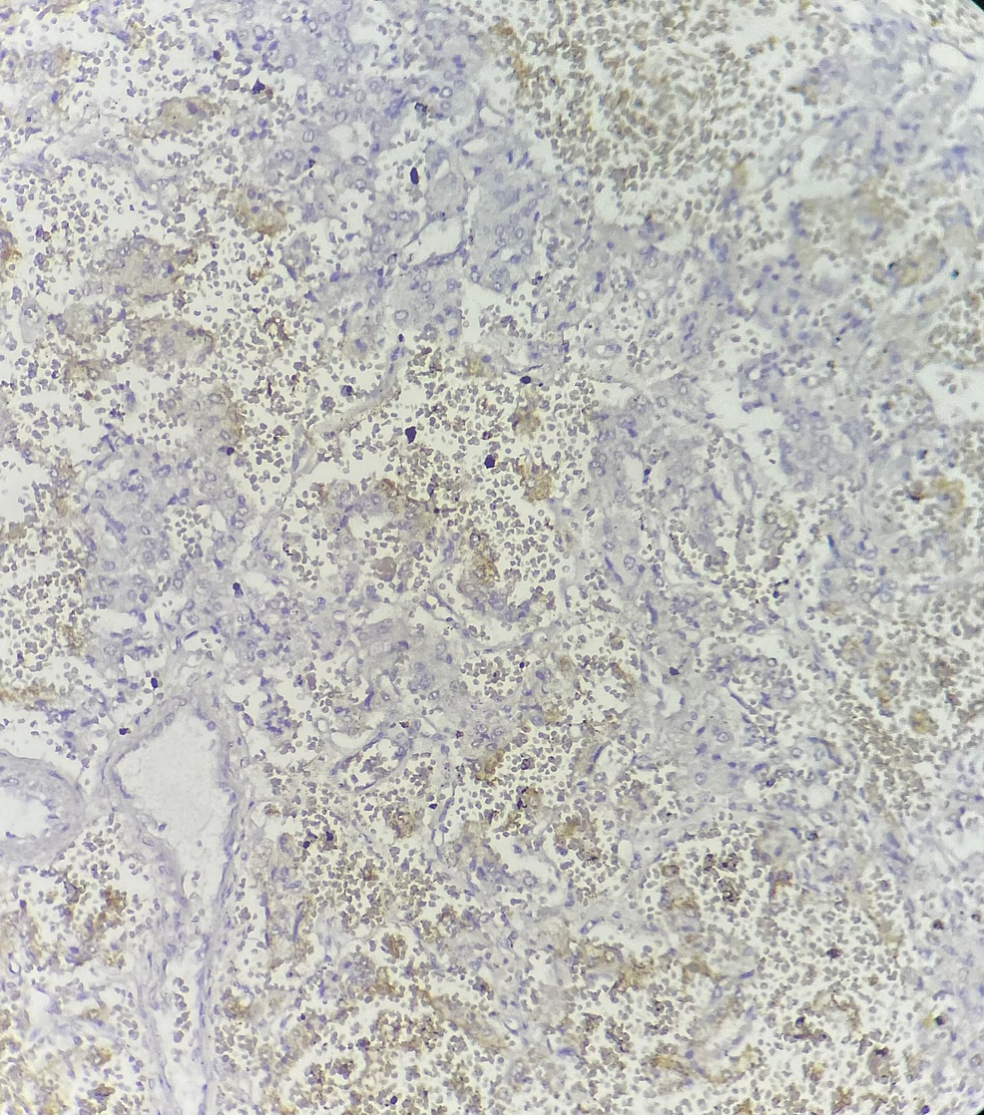
The immunohistochemistry analysis of paraganglioma showing a weak marking of tumor cells for Ki67 estimated at 2% Hematoxylin and eosin (H&E) stain x100

Immunochemistry staining with Ki67 revealed a weak marking of tumor cells estimated at 2% (Figure 5).

The patient's postoperative recovery was unremarkable including refractory hypotension, and she was discharged on the sixth postoperative day. After a follow-up of six months, the patient's hypertensive peaks resolved, and she gave birth at 37 weeks of amenorrhea through vaginal delivery without any complications.

## Discussion

Paragangliomas are very rare extra-adrenal tumors that develop from the chromaffin tissue, and their incidence in pregnant women is about 1/50,000 [6]. However, they may be underestimated due to the difficulty to distinguish their symptoms from the more common causes of gravid hypertension (toxemia and preeclampsia) [7]. Paragangliomas can occur sporadically, as in our patient, or as part of hereditary syndromes like Von Hippel-Lindau disease, multiple endocrine neoplasia (MEN) types 2A and 2B, and neurofibromatosis type 1 [8]. In the sporadic form, the condition can occur between the ages of 20 and 50 years with a female-to-male ratio of around 3:1 [9].

Delayed diagnosis of a secreting paraganglioma can have serious consequences for the mother (acute respiratory distress syndrome, pulmonary edema, disseminated intravascular coagulation, death) and the fetus (spontaneous miscarriage, intrauterine growth retardation, fetal death in utero) [10]. The clinical presentations of paragangliomas are usually variable; despite the asymptomatic forms, the diagnosis might be reached based on persistent or paroxysmal hypertensive peaks resistant to usual antihypertensive treatments associated with tachycardia, pallor headaches, and anxiety [11]. Its association with diabetes and other metabolic disturbances is not rare [12]. Based on frequency, in gravid hypertension, the first diagnosis to be considered is preeclampsia. The absence of proteinuria and edema ruled out this diagnosis, knowing that proteinuria does not exclude the diagnosis of pheochromocytoma or secreting paraganglioma [13]. Biological analysis can confirm the diagnosis by measuring catecholamines (noradrenaline, adrenaline, dopamine) and their urinary metabolites (metanephrine, normetanephrine, and vanillylmandelic acid) [14]. Ultrasound, CT, and MRI can help diagnose and guide the surgical planning of paraganglioma by determining its location and its extent of reach to surrounding structures. Ultrasound, which has a sensitivity of 89-97%, shows an oval, solid, well-limited mass with numerous central cystic-like formations but it is technically difficult, especially at the end of pregnancy with a gravid uterus; CT is feasible during the third trimester. However, MRI is the examination of choice during pregnancy, as it is non-irradiating and very sensitive, showing a mass that is round or oval, homogeneous, cystic, or necrotic [15]. In our case, MRI was used to determine the exact size of the mass, its location, and its relation to the surrounding structures.

The diagnosis of certainty is pathological, with an appearance of a huge rounded tumor, encapsulated with a firm, elastic, and highly vascularized consistency. Immunohistochemistry staining confirms the diagnosis. There is no pathological criterion to distinguish between benign and malignant tumors [16].

The treatment of paragangliomas must be decided within a multidisciplinary setting. Complete surgical resection without microscopic residue remains the only curative treatment with a survival rate exceeding 75% at five years [17]. On the one hand, the surgical approach by laparoscopy is nowadays routinely proposed. However, it does not seem to be superior to open surgery in the prevention of hypertensive peaks, because of the creation of a pneumoperitoneum that stimulates the release of catecholamines from the tumor in parallel to the insufflation pressure, which must therefore be kept as low as possible (less than 12 mmHg) [14,15]. On the other hand, laparoscopic surgery brings a net benefit in terms of postoperative pain and the length of hospital stay. Hence, this technique should be preferred, on the condition that it is performed by experienced operators, which was the case with our patient. After 24 weeks of pregnancy, laparoscopy is difficult because of the volume of the uterus. Ideally, a C-section should be scheduled as soon as the fetal lungs have matured [18,19].

The condition is associated with a poor prognosis, with a fetal mortality rate of 55% and maternal mortality of 25%. Early diagnosis during pregnancy and good therapeutic management can reduce fetal mortality to 15% and maternal mortality to 4% [20].

## Conclusions

Retroperitoneal paraganglioma during pregnancy is a rare entity. The diagnosis is usually reached based on the presence of atypical gravid hypertension, accompanied by suggestive clinical signs, or resistance to treatment. It can be confirmed by simple and reliable biological tests. Imaging tests can help with the diagnosis and surgical planning of paraganglioma. Its management represents a real challenge. The maternal and fetal morbidity rate is significant and requires multidisciplinary management at various points of the disease course. The prognosis is poor, especially in cases of late diagnosis. However, thanks to management optimization, the mortality rate has dropped significantly of late. Hence, we recommend that every case of severe hypertension at a young age be taken seriously and explored thoroughly.
